# Supplementary material for: Efficient and reproducible pipelines for spike sorting large-scale electrophysiology data
Source: eLife. 2026 Aug 5;15:RP110170. doi: 10.7554/eLife.110170 (PMC13441294; doi:10.7554/eLife.110170)
Supplement: Peer review history

# Editor report: eLife Assessment

**DOI:** 10.7554/eLife.110170.3.sa0

**Authors:** Lisa M Giocomo

**Affiliations:** Howard Hughes Medical Institute, Stanford University School of Medicine United States

**Keywords:** Compelling, Valuable

This study presents a **valuable** and well-documented computational pipeline for the scalable analysis and spike sorting of large extracellular electrophysiology datasets, with particular relevance for high-density recordings such as Neuropixels. The authors demonstrate the pipeline's utility for benchmarking spike sorter performance and evaluating the effects of data compression, supported by thorough testing, clear figures, and openly available code. The workflow is reproducible, portable, and practical, providing concrete guidance on computational cost and runtime. Overall, the evidence supporting the pipeline's performance and output quality is **compelling**, and this work will be of broad interest to the systems neuroscience community.

# Referee report: Reviewer #1 (Public review)

**DOI:** 10.7554/eLife.110170.3.sa1

Summary:

Extracellular electrophysiology datasets are growing in both number and size, and recordings with thousands of sites per animal are now commonplace. Analyzing these datasets to extract the activity of single neurons (spike sorting) is challenging: signal to noise is low, the analysis is computationally expensive, and small changes in analysis parameters and code can alter the output. The authors address the problem of volume by packaging the well-characterized SpikeInterface pipeline in a framework that can distribute individual sorting jobs across many workers in a compute cluster or cloud environment. Reproducibility is ensured by running containerized versions of the processing components.

The authors apply the pipeline in two important examples. The first is a thorough study comparing the performance of two widely used spike-sorting algorithms (Kilosort 2.5 and Kilosort 4). They use hybrid datasets created by injecting measured spike waveforms (templates) into existing recordings, adjusting those waveforms according to the measured drift in the recording. These hybrid ground truth datasets preserve the complex noise and background of the original recording. Similar to the original Kilosort 4 paper, which uses a different method for creating ground truth datasets that include drift, the authors find Kilosort 4 significantly outperforms Kilosort 2.5. The second example measures the impact of compression of raw data on spike sorting with Kilosort 4, showing that accuracy, precision, and recall of the ground truth units is not significantly impacted even by lossy compression. As important as the individual results, these studies provide good models for measuring the impact of particular processing steps on the output of spike sorting.

Strengths:

The pipeline uses the Nextflow framework, which makes it adaptable to different job schedulers and environments. The high-level documentation is useful, and the GitHub code is well organized. The two example studies are thorough and well-designed and address important questions in the analysis of extracellular electrophysiology data.

Weaknesses:

There are no major weaknesses in the revised manuscript. While no data analysis pipeline can cover the needs of all experiments, the authors have added and significant flexibility in the pipeline. Even experimenters who might opt for a simpler pipeline will benefit from this work as a model.

# Referee report: Reviewer #2 (Public review)

**DOI:** 10.7554/eLife.110170.3.sa2

Summary:

This work presents a reproducible, scalable workflow for spike sorting that leverages parallelization to handle large neural recording datasets. The authors introduce both a processing pipeline and a benchmarking framework that can run across different computing environments (workstations, HPC clusters, cloud). Key findings include demonstrating that Kilosort4 outperforms Kilosort2.5 and that 7× lossy compression has minimal impact on spike sorting performance while substantially reducing storage costs.

Strengths:

(1)Extremely high-quality figures with clear captions that effectively communicate complex workflow information.

(2) Very detailed, well-written methods section providing thorough documentation.

(3) Strong focus on reproducibility, scalability, modularity, and portability using established technologies (Nextflow, SpikeInterface, Code Ocean)

(4) Pipeline publicly available on GitHub with documentation.

(5) Clear cost analysis showing ~$5/hour for AWS processing with transparent breakdown.

(6) Good overview of previous spike sorting benchmarking attempts in the introduction

(7) Practical value for the community by lowering barriers to processing large datasets.

Weaknesses

No significant weaknesses. The authors have responded to all my review critiques and suggestions.

# Referee report: Reviewer #3 (Public review)

**DOI:** 10.7554/eLife.110170.3.sa3

Summary:

The authors provide a highly valuable and thoroughly documented pipeline to accelerate the processing and spike sorting of high-density electrophysiology data, particularly from Neuropixels probes. The scale of data collection is increasing across the field, and processing times and data storage are a growing concern. This pipeline provides parallelization and benchmarking of performance after data compression that helps address these concerns. The authors also use their pipeline to benchmark different spike sorting algorithms, providing useful evidence that Kilosort4 performs the best of out the tested options. This work, and the ability to implement this pipeline with minimal effort to standardize and speed up data processing across the field, will be of great interest to many researchers in systems neuroscience.

Strengths:

The paper is very well written and clear. The accompanying GitHub and ReadTheDocs are well organized and thorough. Benchmarks are exceptionally well applied to support the authors' claims, and it is clear that the pipeline has been very thoroughly tested and optimized by users at the Allen Institute for Neural Dynamics. The pipeline incorporates existing software and platforms that have also been thoroughly tested (such as SpikeInterface), so the authors are not reinventing the wheel, but rather putting together the best of many worlds. In the latest revision, the authors add a nice analysis showing that compression mostly affects the lowest SNR units. This is a great contribution to the field and it is clear the authors have put a lot of thought into making the pipeline as accessible as possible.

Weaknesses:

None noted. The authors have addressed all previous questions and requests for clarification.

# Author comment: Author response

**DOI:** 10.7554/eLife.110170.3.sa4

**Authors:** Alessio Paolo Buccino, Arjun Sridhar, David Feng, Karel Svoboda, Josh H Siegle

**Affiliations:** Allen Institute for Neural Dynamics Seattle United States; Allen Institute for Neural Dynamics Seattle United States; Allen Institute for Neural Dynamics Seattle United States; Allen Institute for Neural Dynamics Seattle United States; Allen Institute for Neural Dynamics Seattle United States

The following is the authors’ response to the original reviews.

**Reviewer #1 (Public review):**

Weaknesses:

The pipeline is very complete, but also complex. Workflows (optimal artifact removal, best curation for data from a particular brain area or species) will vary according to experiment. Therefore, a discussion of the adaptability of the pipeline in the “Limitations” section would be helpful for readers.

We added a dedicated paragraph in the Discussion section under “Limitations” focusing explicitly on the adaptability and flexibility of the pipeline. Furthermore, we took this feedback as an opportunity to make the pipeline itself significantly more modular and customizable with the most recent release (v1.2.0: here).

**Reviewer #1 (Recommendations for the authors):**

(1) In the description of the Phase-shift correction (Line 166-167): The current text reads “As a result, different groups of channels are sampled asynchronously.” A better description would be: “Sample times for different groups of channels are offset in time by a known amount.”

We replaced the phrase in the manuscript text with the suggested formulation.

(2) Figure 5 and description of the benchmarking overview (Line 326-336): How were spike trains (times) selected for the injected ground truth units? What was the range of firing rates?

All injected spike trains were generated as independent Poisson processes featuring a mean firing rate of 15 Hz. We have now incorporated this explicitly into the main text to clarify the ground-truth injection process.

(3) Figure 6, panel b: Are the gray points in the raster the original spikes in the test recording? From the pattern, it looks like there are 8 recovered ground truth units. Were the other 2 undetected by either sorter?

That is correct; the two remaining units were undetected by both sorters. To clear up any confusion, we updated the caption for Figure 6 to state: “Note that spikes undetected by any of the sorter are not shown in the plot.”

(4) Figure 7, panel c: Are all units returned from KS included in these distributions? (i.e., regardless of the KS refractory metric calculated by the sorter) - it would be useful to add that detail to the caption. It would also be helpful for panel C to include a total unit count from the two sorters... Also, since there are multiple ways to calculate the refractory period contamination, it would be good to state the calculation used here.

Because we rely directly on the hybrid ground-truth for accurate validation, we included all raw units returned by Kilosort for this specific analysis. We have explicitly added a note detailing this to the caption. Panel C does report the total raw unit count returned by the two sorters (*N* = 3046 for KS2.5; *N* = 3652 for KS4).

Additionally, to clarify the evaluation procedure, we appended the following statement to the main text: “For all results, we perform spike train comparisons and compute performance metrics as defined in (Buccino et al. 2020), using all units returned by the spike sorter (without any sorterspecific curation).”

(5) Comments about the pipeline:

The paper clearly demonstrates the immense utility of the pipeline in the authors’ work. I did some testing to try to understand its adaptability to workflows at my institution.

I tested the pipeline on our local cluster running LSF. I’ve worked on a similar pipeline using Nextflow to automate ephys analysis with the same sorters. Questions that came up for me that would be usefully addressed in the ’Limitations’ section:

(i) Is the pipeline meant to be run only in total? In particular, is it possible to start with preprocesseddata? (aind-ephys-preprocessing/code/params.json does not appear to include any means to turn off filtering, for example). Is the pipeline meant to be run only in total? In particular, is it possible to start with preprocessed data? (aind-ephys-preprocessing/code/params.json does not appear to include any means to turn off filtering, for example).

To accommodate users who wish to run only parts of the workflow or use external preprocessing setups, we have refactored the codebase to support a custom preprocessing pipeline option. This makes it possible to turn off standard filtering or inject custom workflows.

(ii) For debugging purposes, is there a means to go from preprocessing or sorting to result collection,so that interim results can be interpreted even when some steps of the pipeline aren’t working?

The pipeline is designed to be a spike sorting pipeline, so the spike sorting step cannot be skipped. However, we have rewritten the post-sorting architecture to make it highly lightweight and fault-tolerant. The postprocessing step now only requires the random spikes and templates computation and downstream steps have been update to accomodate this lightweight option. As an example, if no quality metrics are computed, the curation step will be skipped. The visualization and QC steps also required updates to be tolerant to missing extensions. This required coordinate updates across several components:

Postprocessing: PR #12

Curation: PR #13

Visualization: PR #21

Quality Control: PR #20

(iii) If these options to skip processes and output data ’partway’ are available, it would be great toadd that to the documentation.

We have fully updated our online documentation for v1.2.0 (release notes: here), introducing a brandnew “Customization” guide page that comprehensively explains how to construct and provide custom preprocessing and postprocessing strategies, as well as how to integrate a new spike sorter in the pipeline: here

**Reviewer #2 (Public review):**

Summary:

This work presents a reproducible, scalable workflow for spike sorting that leverages parallelization to handle large neural recording datasets. The authors introduce both a processing pipeline and a benchmarking framework that can run across different computing environments (workstations, HPC clusters, cloud). Key findings include demonstrating that Kilosort4 outperforms Kilosort2.5 and that 7× lossy compression has minimal impact on spike sorting performance while substantially reducing storage costs.

Strengths:

(1) Extremely high-quality figures with clear captions that effectively communicate complex workflow information.

(2) Very detailed, well-written methods section providing thorough documentation.

(3) Strong focus on reproducibility, scalability, modularity, and portability using established technologies (Nextflow, SpikeInterface, Code Ocean).

(4) Pipeline publicly available on GitHub with documentation.

(5) Clear cost analysis showing ~$5/hour for AWS processing with transparent breakdown.

(6) Good overview of previous spike sorting benchmarking attempts in the introduction.

(7) Practical value for the community by lowering barriers to processing large datasets.

Weaknesses:

No significant weaknesses were identified, although it is noted that the limitations section of the discussion could be expanded.

We thank the reviewer for their constructive feedback on our manuscript.

**Reviewer #2 (Recommendations for the authors):**

The authors could discuss why 2.25 bps is the “lowest supported” level and whether more aggressive compression could be achieved with custom approaches, potentially exploring where performance breakdown occurs.

The 2.25 bits-per-sample (bps) limit is an inherent constraint of the WavPack lossy compression library itself. While more aggressive, domain-specific, or custom compression schemes could be explored, we focused on WavPack due to its native support in modern neurophysiology ecosystems and its excellent performance in our prior simulated benchmarks (Buccino et al. 2023). We agree that using this hybrid benchmarking framework to explore alternative compression configurations is a highly valuable avenue for future work. We have added the following text to the Discussion: “The benchmarking pipeline will continue to develop as an open evaluation framework, enabling transparent and reproducible comparisons of spike sorting and preprocessing methods across the community. As one example, the work on lossy compression could be extended with additional codecs and parameter settings, exploiting our ability to read out spike sorting degradation directly from the hybrid ground truth spike times.”

(2) The limitations section would benefit from expansion to include: (i) discussion of how simulated data limitations may affect generalization of benchmarking results to real neural data, and (ii) clarification of the effort required to add new spike sorters, including configuration complexities for coordinating Nextflow processes beyond simple SpikeInterface integration.

We have expanded the Discussion section to address both items:

(i) We added a paragraph detailing the specific limitations of hybrid ground-truth datasets (e.g., how idealized template injection might miss extreme multi-unit overlapping dynamics or nonstationary noise properties found in real tissue).

(ii) We added a structural overview section clarifying the workflow complexity, detailing exactly what steps are required to map a new spike sorter into a Nextflow execution processes beyond its baseline addition to Spike Interface.

(3) The authors should clarify the terminology of “hypothetical experiment” in the introduction to improve reader comprehension.

We have removed the word hypothetical from the introduction to ground the explanation more directly.

(4) The cost analysis could be improved by making it clearer whether “runtime” refers to wall-clock vs. total parallel compute time.

We mean wall-clock time. While total parallel compute time aggregated across cloud workers remains roughly identical to the overall sequential execution on a lone cloud instance, cluster parallelization slashes the wall-clock time drastically. We have updated the text to explicitly state that reported runtimes represent wall-clock time.

(5) The authors could address the Nextflow Java dependency limitation by discussing containerized execution options (Docker/Singularity) as a solution, while noting relevant HPC system restrictions.

We have updated the text to mention the official pre-built Nextflow container images as an elegant workaround for environments where local Java installations are blocked or restricted: “However, one option to bypass installation issues is to run the main pipeline script in container images packaged with Nextflow (https://hub.docker.com/r/nextflow/nextflow).”

(6) Figure 8 analysis would be strengthened by explicitly noting that compression effects are more substantial for lower-accuracy units, suggesting better preservation of higher SNR units.

We appreciate this insight. To evaluate this systematically, we generated a new supplementary figure (Figure S3) which shows sorting performance during lossy compression as a function of the Signal-to-Noise Ratio (SNR) of ground truth units. The plot demonstrates that for Neuropixels 2.0 recordings, the slight drop in sorting accuracy is indeed heavily concentrated among low-SNR units. We have integrated this observation into the Results section.

**Reviewer #3 (Public review):**

(1) Could the authors please expand on the statement on line 274, that processing their test dataset serially “on a single GPU-capable cloud workstation... would take approximately 75 hours and cost over 90 USD.” How were these values calculated? I was a bit surprised that this is a 4-fold slowdown from their pipeline, but only increases the cost by 1.35x... More context on why this is, and maybe some context on what a g4dn.4xlarge is compared to the other instances, might help.

We have expanded the cost analysis section in the manuscript methods to explain these figures explicitly. The serial run relies on a single continuous, higher-tier GPU workstation instance (g4dn.4xlarge) running uninterrupted for 75 hours.

Our distributed pipeline, by contrast, dynamically provisions CPU-only instances to process chunked preprocessing steps concurrently, then spins up short-lived GPU spot instances only when Kilosort executes. While this parallel execution compresses the overall wall-clock time by over 4-fold, the cost is only moderately reduced because the CPU-only instances with many parallel processing cores are only slightly less expensive than GPU instances.

(2) One of the most commonly used preprocessing pipelines for Neuropixels data is the CatGT/ecephys pipeline from the developers of SpikeGLX at Janelia. It may be worth commenting very briefly... on how the preprocessing steps available in this pipeline compare to the steps available in CatGT. For example, is “destriping” similar to the “-gfix” option in catGT to remove high-amplitude artifacts?

We have added a section drawing direct comparisons to CatGT preprocessing workflows. We explicitly clarify that our phase-shift correction performs the exact same function as CatGT’s Tshift. We also point out that while our current version lacks a direct equivalent to CatGT’s saturation removal feature (-gfix), this capability is scheduled for incorporation in our upcoming pipeline release.

(3) Why are there duplicate units (line 194), and how often is this an issue? I understand that this is likely more of a spike sorter issue than an issue with this pipeline, but 1-2 sentences elaborating why might be helpful for readers.

Duplicate units are primarily an artifact of template-matching sorting routines (such as Kilosort), which can occasionally split a single biological neuron into multiple overlapping spatial templates or over-extract templates in highly active channel regions. We have added two clarifying sentences explaining this phenomenon in the text: “Next, duplicated units, that can arise when using template-matching methods if different templates are consistently fit to the same spikes, are removed based on the fraction of overlapping spikes.”

Customizability of cluster curation parameters It seems from the parameter files on GitHub that the cluster curation parameters are customizable - correct? If so, it may be worth explicitly saying so in the curation section of the text... A presence ratio of >0.8 could be particularly problematic for some recordings (e.g. state transitions, behavior specific cells).

(4) Yes, they are completely customizable. We agree that a rigid presence ratio cutoff of 0.8 would erroneously discard highly valid units that are modulated by specific behavioral states, or are active only during sleep vs. wake cycles. We have explicitly added text in the Curation section clarifying that all quality metric thresholds can be modified by the user: “Units are tagged as passing a *default_qc* when they satisfy the following criteria based on quality metrics thresholds. Thresholds can be user defined, and these are the default”.

(5) The axis labels in Figures 3d-e are too small to see, and Figure 3d would benefit from a brief description of what is shown.

We have updated the figures with enlarged, high-visibility axis labels and expanded the caption of Figure 3d to clearly describe the visualization.

Figure 4 labels (“neural” vs “passing QC”) (6) What is the difference between “neural” and “passing QC” in Figure 4?

We have updated the figure caption for Figure 4 to include an explicit cross-reference to the Curation methodology section, which defines the strict quantitative boundary between raw neural classification and formal automated QC passage.

(7) I understand the current paper is focused on spike data... but I am curious about the NP2.0 probes that save data in wideband. Does the lossy compression negatively affect the LFP data? Is software filtering applied for the spike band before or after compression?

Compression is applied to the raw streams prior to any secondary downstream software processing. For Neuropixels 1.0, compression is executed strictly on the action potential (AP) stream. For Neuropixels 2.0, compression operates directly on the unified wide-band data stream.

Software filtering to separate bands is conducted post-decompression, as captured in our baseline workflow definitions (e.g., WavPack compression → decompression → preprocessing → Kilosort4). To clarify this, we added the following text: “In all cases, compression was applied before any preprocessing took place. For Neuropixels 1.0, we compressed the AP stream only. For Neuropixels 2.0, we compressed the full wide-band data.”

Because LFP signals possess inherently smooth continuous dynamics across both space and time, they are much more amenable to lossless or near-lossless compression. Thus, the minor losses introduced by lossy compression are overwhelmingly localized to high-frequency spike band features, leaving LFP components virtually unaffected.
